# Deleterious effects of phosphate on vascular and endothelial function via disruption to the nitric oxide pathway

**DOI:** 10.1093/ndt/gfw252

**Published:** 2016-07-22

**Authors:** Kathryn K Stevens, Laura Denby, Rajan K Patel, Patrick B Mark, Sarah Kettlewell, Godfrey L Smith, Marc J Clancy, Christian Delles, Alan G Jardine

**Affiliations:** 1 BHF Cardiovascular Research Centre, Institute of Cardiovascular and Medical Sciences, University of Glasgow, Glasgow, UK; 2 The Renal Transplant Unit, Western Infirmary, (Now based at The Queen Elizabeth University Hospital) Glasgow, UK

**Keywords:** cardiovascular risk, chronic kidney disease, endothelial function, nitric oxide, phosphate

## Abstract

**Background:**

Hyperphosphataemia is an independent risk factor for accelerated cardiovascular disease in chronic kidney disease (CKD), although the mechanism for this is poorly understood. We investigated the effects of sustained exposure to a high-phosphate environment on endothelial function in cellular and preclinical models, as well as in human subjects.

**Methods:**

Resistance vessels from rats and humans (± CKD) were incubated in a normal (1.18 mM) or high (2.5 mM) phosphate concentration solution and cells were cultured in normal- (0.5 mM) or high-phosphate (3 mM) concentration media. A single-blind crossover study was performed in healthy volunteers, receiving phosphate supplements or a phosphate binder (lanthanum), and endothelial function measured was by flow-mediated dilatation.

**Results:**

Endothelium-dependent vasodilatation was impaired when resistance vessels were exposed to high phosphate; this could be reversed in the presence of a phosphodiesterase-5-inhibitor. Vessels from patients with CKD relaxed normally when incubated in normal-phosphate conditions, suggesting that the detrimental effects of phosphate may be reversible. Exposure to high-phosphate disrupted the whole nitric oxide pathway with reduced nitric oxide and cyclic guanosine monophosphate production and total and phospho endothelial nitric oxide synthase expression. In humans, endothelial function was reduced by chronic phosphate loading independent of serum phosphate, but was associated with higher urinary phosphate excretion and serum fibroblast growth factor 23.

**Conclusions:**

These directly detrimental effects of phosphate, independent of other factors in the uraemic environment, may explain the increased cardiovascular risk associated with phosphate in CKD.

## INTRODUCTION

Hyperphosphataemia is an independent risk factor for accelerated cardiovascular disease (CVD) in chronic kidney disease (CKD) [1–7]. CVD is more prevalent in CKD patients than in the general population and is the biggest single contributor to markedly reduced life expectancy, with a predominance of sudden death and heart failure, rather than coronary artery disease [8–10]. The relationship between conventional CV risk factors and CV events in CKD has proved difficult to define and focus has turned to non-traditional risk factors and therapeutic targets, such as hyperphosphataemia [5, 11–14]. Based on observational data linking elevated phosphate with adverse outcomes, most patients with advanced CKD receive phosphate-lowering treatment with oral phosphate binders, although we lack specific evidence on the cardiac and survival benefit of this therapeutic strategy [4, 6, 15–23]. There is also strong evidence that ‘normal’ phosphate levels at the upper limit of the reference range increase the CV risk in other populations, including recipients of a renal transplant, healthy individuals or individuals without CKD but with pre-existing CV disease [12, 14, 22, 24]. One explanation may be that serum phosphate is a poor measure of total body phosphate, as phosphate is largely an intracellular anion. This is reflected in CKD, where the CV risk is increased before phosphate levels rise above the reference range—phosphate seems to be a continuous risk factor, although a phosphate level within the reference range is largely ignored in CKD [15].

Phosphate levels can be easily manipulated. Current means of modification may be suboptimal in terms of risk reduction and because there is limited information about phosphate's mechanism of action as a CV risk factor, it has proved difficult to determine the best approach to altering phosphate levels [25]. The established view is that elevated phosphate contributes to vascular calcification and thus CVD [26–28]. However, alternative mechanisms include direct effects of phosphate on endothelial function, and effects mediated by regulators of phosphate homoeostasis, including fibroblast growth factor 23 (FGF-23), a phosphaturic hormone implicated in cardiac hypertrophy, another common feature of CKD [4, 28–31]. In reality it is likely to be a combination of these effects that contributes to CV risk in CKD. Defining the isolated actions of elevated phosphate independent of other abnormalities of the uraemic environment is challenging.

Emerging evidence supports direct effects of phosphate on vascular function. Shuto *et al.* demonstrated impaired endothelial dysfunction in healthy males following a single high-phosphate content meal, consistent with an acute effect of phosphate [32]. In the same study, endothelium-dependent vasodilatation was impaired in rat aortic rings exposed acutely to high-phosphate concentration. In a preclinical rat model of adenine-induced CKD, aortic rings from rats fed a low-phosphate diet for 16 days exhibited significantly improved endothelium-dependent vasodilatation compared with animals fed standard rat chow [28]. In cells in culture, there is evidence of disruption of the nitric oxide (NO) pathway upon exposure to both a low- and high-phosphate environment, although the authors attributed this to the concomitant reduction in intracellular calcium [33].

Overall, these elegant studies support direct, acute effects of phosphate on vascular cells and function but they do not explore more chronic effects that are relevant to CKD, nor the mechanisms or potential for new therapeutic approaches.

Endothelial dysfunction is a feature of CKD and may contribute to CV risk, probably via disruption of the NO pathway [32, 34, 35]. We hypothesized that phosphate has direct effects on endothelial function via dysregulation of the NO pathway and this explains the association between phosphate and CV risk. We show, for the first time, in translational cell to animal to human studies, the direct effects of prolonged exposure to elevated phosphate concentration on vascular and endothelial function, specifically examining the isolated actions of elevated phosphate independent of other effects of the uraemic environment.

## MATERIALS AND METHODS

For detailed materials and methods, see Supplementary Methods online.

### Vessel studies

Rat mesenteric vessels were utilized and human resistance vessels came from subcutaneous abdominal fat, removed prior to the use of diathermy or the harmonic scalpel, at the beginning of surgery. Twelve-week-old male Wistar-Kyoto rats were sacrificed in accordance with the Animals Scientific Procedures Act 1986. Live kidney donors (LKDs) undergoing nephrectomy for living kidney donation and patients with CKD undergoing live donor renal transplant were identified. Blood samples were collected the day prior to surgery. All myography experiments were performed on a four-chamber wire myograph. Experiments were conducted after storage of the vessels at 4°C in a normal- (1.18 mM) or high-phosphate (2.5 mM) concentration solution for 16 h (Supplementary data, Table S1).

#### Statistical analysis

All responses are expressed as mean ± standard error of the mean (SEM) and comparison made between the areas under the curve (AUC) of groups with Student's *t* test, unless otherwise stated. For comparisons between maximal vasodilation or contractile responses, an unpaired Student's *t* test (rat vessels) or an ANOVA with Tukey's *post hoc* analysis (human vessels) was used. The median L100 (rat vessels only) and the median vessel lengths were compared with a Mann–Whitney *U* test. Statistical analysis was performed in SPSS v 19 (IBM, Armonk, NY, USA).

### Cell culture

Cell media was either of standard phosphate concentration (0.5 mM) or custom formulated (phosphate concentration 3 mM). Cells were grown in standard phosphate concentration medium until they reached 90% confluence. At the first passage, they were divided into standard and high-phosphate concentration cells and grown in the appropriate media from that point.

### Clinical study

This was a single-blind crossover study with healthy volunteers without CKD. Volunteers were screened to ensure they were ‘healthy’ prior to inclusion. Each participant attended three visits. At visit one, patients were randomized to receive either 500 mg phosphate or 1000 mg of lanthanum three times daily for 2 weeks. The participants knew which tablet they were taking but the investigator did not. After 2 weeks, patients attended for the second visit. After a further 2-week washout period, patients received the other drug for 2 weeks, before attending for the final visit. Supplementary data, Figure S1 illustrates the study visit protocol. At each visit, the same measures were taken and pill counts were performed to assess compliance.

Serum and plasma samples were sent to the laboratory and blood was stored at −80°C for FGF-23, cyclic guanosine monophosphate (cGMP) and vitamin D analysis. Prior to each visit, a 24 h urine collection was obtained.

Endothelial function was measured using flow-mediated dilatation (FMD) and measurements were standardized [36]. All recordings and analyses were performed by one investigator. Vascular stiffness was measured using the SphygmoCor^®^ Vx system and standardized [37].

#### Statistical analysis

Data were assessed for normality. Univariate and multivariate linear regression models were constructed with change in FMD from baseline as the measure of outcome. The crossover nature of the study utilizing the same patients for each arm was taken into account and randomization order was included in the model. One-third of the images were randomly selected and reanalysed to test reproducibility and intra-observer variability.

## RESULTS

We exposed cells and vessels to two phosphate concentrations, normal and high (0.5 and 3 mM in cell lines and 1.18 and 2.5 mM in vessels). For context, 0.5 mM is the standard concentration of phosphate in the conventional cell culture medium suited to the cells utilized, 0.8–1.4 mM is the normal plasma range in humans, whilst 2.5–3 mM is at the extreme of the CKD spectrum.

### Effect of phosphate on the function of rat and human vessels *in vitro*

**FIGURE 1:**
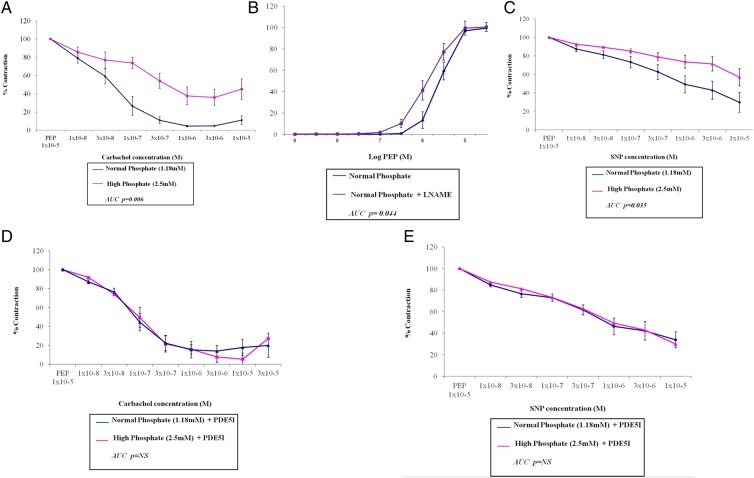
Rat mesenteric vessels incubated in a high-phosphate concentration (2.5 mM) solution have impaired endothelium-dependent and -independent vasodilatation, an effect that can be reversed when the vessels are co-incubated with a phosphodiesterase-5 inhibitor (PDE5I), zaprinast. Vessels were incubated for 16 h in either a normal (1.18 mM) or high (2.5 mM) phosphate concentration solution and all responses are expressed as mean ± SEM. (**A** and **C**) Vasodilatation to increasing concentrations of carbachol or SNP, respectively, expressed as a % of maximal contraction with PEP 1 × 10^−5^ M. (**B**) Contraction with PEP in the presence and absence of L-NAME. (**D** and **E**) Vasodilatation to increasing concentrations of carbachol or SNP, respectively, in the presence of zaprinast (PDE5I) expressed as a % of maximal contraction with PEP 1 × 10^−5^ M. *n* = 10–13 vessels (see Table 1).

Table 1 shows the numbers of rat mesenteric vessels used and their normalized internal diameter (L100), which was not significantly different between the groups. In phenylephrine (PEP) pre-constricted vessels, the endothelium-dependent vasodilator carbachol was less effective in high- compared with normal-phosphate conditions (Figure 1A; maximum vasodilatation 64 ± 9% versus 95 ± 1%; P < 0.001). When we assessed basal NO production using the NO synthase inhibitor L-NAME (LN), we found that in normal-phosphate vessels, LN shifted the concentration–response curve to PEP significantly to the left compared with contraction in the absence of LN (Figure 1B). In high-phosphate conditions, no significant shift was observed (EC50 + LN: 4.5 ± 2.1 × 10^−6^ M versus EC50-LN: 5.3 ± 2.8 × 10^−6^ M; data not shown). To assess endothelium-independent vasodilatation, we used the NO donor sodium nitroprusside (SNP). Vasodilatation to SNP was reduced in high- versus normal-phosphate conditions (Figure 1C; maximal vasodilatation 45 ± 22% versus 73 ± 26; P = 0.02). Adding zaprinast, a phosphodiesterase (PDE5) inhibitor (1 × 10^−5^ M for 1 h), to increase levels of cGMP reversed the effects of high phosphate, improving vasodilator responses to carbachol and SNP (Figure 1D and E).

**Table 1:** Comparison of numbers of vessels studied for each drug and the corresponding size of the vessels

	Incubated in standard phosphate	Incubated in high phosphate	P
Phenylephrine			
Number	12	10	
L100	366.5 (292.7–422.3)	401.6 (226.3–525.4)	NS
Carbachol			
Number	12	10	
L100	366.5 (292.7–422.3)	401.6 (226.3–525.4)	NS
L-Name			
Number	12	10	
L100	366.5 (292.7–422.3)	401.6 (226.3–525.4)	NS
SNP			
Number	11	9	
L100	391.9 (311.2–504.3)	396.9 (267.5–525.3)	NS
Carbachol and PDE5I			
Number	10	13	
L100	387.2 (324.2–412.1)	384.1 (341–411.6)	NS
SNP and PDE5I			
Number	10	13	
L100	387.2 (324.2–412.1)	384.1 (341–411.6)	NS

Number is the number of vessels for which a complete concentration response curve was obtained. L100 is the normalized internal diameter of the vessels with comparison by the Mann–Whitney *U* test.

PDE5I, phosphodiesterase-5 inhibitor (zaprinast); SNP, sodium nitroprusside; NS, not significant.

These data are consistent with impaired endothelium-dependent and -independent vasodilation and reduced basal NO production in high-phosphate conditions, effects that are potentially reversible.

To determine whether these findings could be translated to humans, we studied resistance vessels from LKDs and patients with CKD stage 5 (Table 2). Vessels were obtained at the time of live donor nephrectomy or at the time of transplantation. Comparisons were made between subjects with and without CKD. The former had higher serum phosphate and FGF-23 levels. Eighty-nine per cent (*n* = 8) received a phosphate binder. No patient was taking drugs that interfere with NO metabolism. Table 3 details the vessels used.

**Table 2:** Demographics of the study population

Parameter	Live donor	CKD	P
Age (years)	48.8 ± 10.2	44.6 ± 14.5	NS
Male sex (%)	64	44	
Systolic BP	134 ± 16	134 ± 24	NS
BMI	**29.5 ± 3.6**	**25.4 ± 3.4**	**0.02**
Dialysis (%)	0	44	
eGFR (mL/min/1.73 m^2^)	**95.8 ± 21**	**8.0 ± 3.0**	**<0.001**
Phosphate (mmol/L)	**1.1 ± 0.1**	**1.7 ± 0.6**	**0.003**
Adjusted calcium (mmol/L)	2.4 ± 0.1	2.5 ± 0.1	NS
FGF-23 (RU/mL)	**45.8 (40.5–51.7)**	**1644 (1135.7–1750.4)**	**<0.001**
On any medication (%)	18	100	
Calcium-containing phosphate binder (%)	0	33	
Non-calcium-containing phosphate binder (%)	0	67	
Alfacalcidol (%)	0	78	
Cinacalcet (%)	0	0	
Antihypertensive (%)	18	89	

BP, blood pressure; BMI, body mass index; NS, not significant.

Live donor, vessels from live donor nephrectomy; CKD, vessels from recipients of live kidney transplant. Demographics for the patients whose vessels were used are shown in columns two to four. Live donor, *n* = 14 and CKD, *n* = 14.

**Table 3:** Comparison of numbers of vessels studied for each patient group

	CKD		Live donor		
	Normal	High	Normal	High	
Length	4.76 ± 0.36	4.52 ± 0.49	4.59 ± 0.35	4.51 ± 0.44	
Diameter	494.3 (339–700)	460.9 (340–792)	534.2 (286–879)	438.4 (297–684)	
Phenylephrine^a^	12	9	13	12	
Carbachol^a^	12	9	11	12	
SNP^a^	12	9	13	12	

Measurements are in microns.

The number of vessels in which a complete concentration response curve was obtained for each drug and the corresponding size of the vessels are presented. Comparison was made with a one-way ANOVA.

^a^These values represent the number of vessels used (*n*).

**FIGURE 2:**
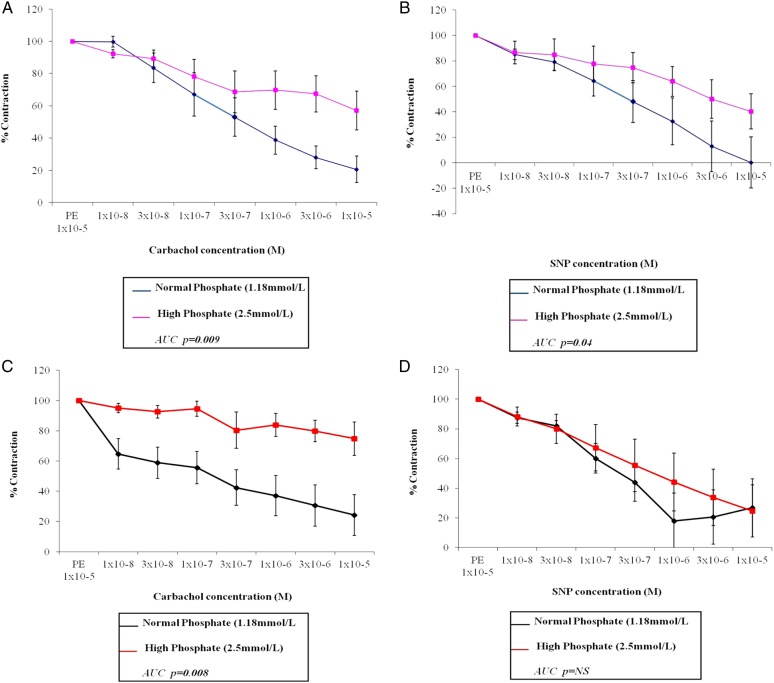
Human resistance vessels from patients with (**C**) and without (**A**) CKD incubated in a high-phosphate concentration (2.5 mM) solution have impaired endothelium-dependent vasodilatation. Endothelium-independent vasodilatation is impaired in vessels from humans without CKD (**B**) but preserved in those with CKD (**D**). Vessels were incubated for 16 h in either a normal (1.18 mM) or high (2.5 mM) phosphate concentration solution and all responses are expressed as mean ± SEM. (A and C) Vasodilatation to increasing concentrations of carbachol expressed as a % of contraction with PEP 1 × 10^−5^ M in vessels from living kidney donors without CKD (A) and from patients with CKD stage 5 (C). (B and D) Vasodilatation to increasing concentrations of SNP expressed as a % of contraction with PEP 1 × 10^−5^ M in vessels from living kidney donors without CKD (B) and from patients with CKD stage 5 (D). *n* = 9–14 vessels (see Table 3).

In LKD vessels, in high-phosphate compared with normal-phosphate conditions, vasodilatation to carbachol was impaired (Figure 2A; maximum vasodilatation 42.9 ± 12% versus 79.4 ± 8.2%; P = 0.003). Similarly, high-phosphate LKD vessels vasodilated less well to SNP compared with normal-phosphate vessels (Figure 2B; maximum vasodilatation 59.7 ± 13.8% versus 99.8 ± 20.2%; P = 0.02). Impaired vasodilation with carbachol was also observed in CKD vessels incubated in high- versus normal-phosphate conditions (Figure 2C; maximum vasodilatation 25.3 ± 11.1% versus 75.7 ± 13.6%; P < 0.001).

Interestingly, there was no significant difference in either AUC or maximal relaxation between the CKD and the LKD vessels incubated in normal-phosphate conditions, supporting the notion that the effects of phosphate may be reversible. There was no significant difference in relaxation to SNP between the CKD vessels incubated in high- or normal-phosphate conditions (Figure 2D). In LKD with normal renal function, both endothelium-dependent and -independent vasodilation are impaired, consistent with the effects observed in our preclinical model. In CKD resistance vessels endothelium-dependent vasodilation was blunted in high-phosphate conditions, but when incubated *ex vivo* in a normal-phosphate solution, the relaxation response was similar to that of healthy LKD vessels.

### Effect of phosphate on the NO pathway

Since the data above, in isolated vessels, suggest a direct and reversible effect of phosphate on the endothelium mediated by disruption of both basal and stimulated NO production, we next explored the NO pathway further by examining the effects of phosphate in cell lines.

**FIGURE 3:**
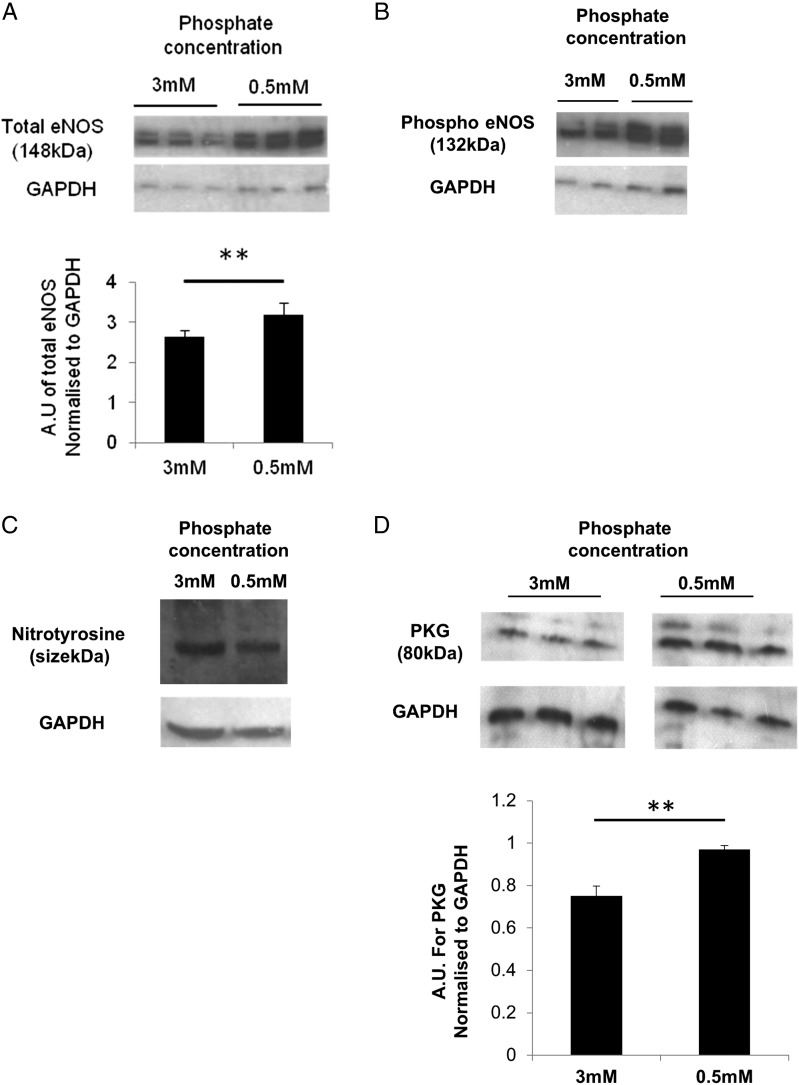
HUVECs express reduced total (**A**) and phospho (**B**) eNOS and increased nitrotyrosine (**C**) when cultured in high-phosphate concentration medium (3 mM). PKG expression is reduced in rat VSMCs cultured in high- compared with normal-phosphate concentration medium. **P < 0.05. HUVECs and VSMCs were cultured in 0.5 mM or 3 mM phosphate concentration medium. Ten µg of protein was fractionated on SDS page gels. Primary antibodies for Total eNOS (1:1000), Phospho eNOS (1:200) (both Cell Signalling Technology), nitrotyrosine (1:500, R & D Systems), PKG (1:200, Enzo Life Sciences) and protein expression normalized to GAPDH. Densitometry was performed. In (A)–(**D**), top panels show representative gel from cells cultured in 0.5 or 3 mM phosphate concentration medium and bottom panels show GAPDH. The panels were all taken from a single gel and each band represents one well from separate six-well plates.

We measured endothelial nitric oxide synthase (eNOS) expression and NO production in human umbilical vein endothelial cells (HUVECs), as well as nitrotyrosine expression to investigate the role of reactive oxygen species (ROS) and free radicals in the high-phosphate environment. Total and phospho eNOS (normalized to GAPDH) and NO levels (data not shown) were significantly reduced in HUVECs cultured in high-phosphate conditions (Figure 3A and B). There was evidence of increased free radical formation in a high-phosphate environment as there was increased expression of nitrotyrosine (Figure 3C).

The smooth muscle cell is vital for a contractile response instigated via an intact NO pathway. In view of the impaired endothelium-independent vasodilatation to SNP observed in our resistance vessels, we measured cGMP levels in vascular smooth muscle cells and the resistance vessels exposed to normal and high-phosphate environments and protein kinase G (PKG), a cGMP-dependent kinase implicated in the relaxation of smooth muscle.

**FIGURE 4:**
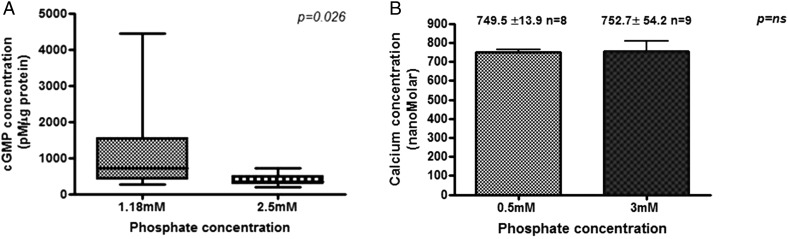
cGMP concentration is significantly reduced in rat vessels incubated for 24 h in a high-phosphate concentration solution (**A**), whereas the intracellular calcium level in HUVECs is unchanged in a high-phosphate environment (**B**). (A) Rat mesenteric resistance vessels were incubated in a 1.18 mM (*n* = 11) or 2.5 mM (*n* = 11) phosphate concentration solution for 24 h. Results are expressed as cGMP pM/µg protein. Analysis was by the Mann–Whitney *U* test. (B) Cells were plated in glass-bottomed plates and cytosolic loading of FURA-2-AM was achieved by incubating the cells with FURA-2-AM. Values are mean ± SEM and are from at least four experiments with the number of cells included indicated by (*n*).

In rat vascular smooth muscle cells (VSMCs), cultured in high-phosphate conditions, PKG expression was significantly reduced (Figure 3D). In rat mesenteric vessels, cGMP was higher in vessels incubated in low- versus high-phosphate conditions (715.6(418–1541) pM/µg versus 330.6 (284.1–500.1) pM/µg; P = 0.0026; Figure 4A). In a high-phosphate environment, in support of our hypothesis that there is disruption in the entire NO pathway from the endothelial cell to the smooth muscle cell, we demonstrate reduced eNOS and PKG and increased nitrotyrosine expression, and reduced NO and cGMP production.

Although these data are consistent with a direct effect of phosphate, we measured intracellular calcium to exclude an indirect effect mediated by reduction in intracellular calcium. In HUVECs, intracellular calcium measurements were not significantly different between cells cultured in high- versus normal-phosphate conditions (Figure 4B; 749.5 ± 13.9 nM versus 752 ± 54.2 nM; P = not significant); rat VSMCs showed the same pattern (228.3 ± 22.0 nM versus 278 ± 22.4 nM).

### Effects of sustained phosphate loading in healthy human volunteers

To determine whether the observations on NO and endothelial function in cellular and preclinical models are of physiological relevance in man, we performed a study of phosphate loading and phosphate binders (lanthanum) in healthy subjects, using FMD to assess endothelial function. Healthy volunteers, rather than CKD patients, were selected initially to allow study of the sole effects of phosphate, independent of the uraemic milieu.

We recruited 19 volunteers: 63% (*n* = 12) were female and the mean age was 42.2 ± 14.3 years (Table 4). At baseline, mean estimated glomerular filtration rate (eGFR) was 102 ± 10 mL/min/1.73 m^2^, serum phosphate level was 1.05 ± 0.18 mmol/L and the fractional excretion of urinary phosphate (FeP) was 14.3 ± 3.4%. Participants were normoglycaemic, with normal mean lipid values. The median FMD was 8.4% (6.2–11.6%) post-cuff inflation and 17.7% (13.4–23.2%) post-GTN spray.

**Table 4:** Participant demographics at baseline, following lanthanum and phosphate

Parameter	Baseline	Post-lanthanum	Post-phosphate	P-value
Age (years)	42.2 ± 14.3			
Male sex	36.8% (*n* = 7)			
BMI	26.0 ± 4.1	26.3 ± 3.9	26.3 ± 3.8	NS
Creatinine (µM)	66.4 ± 6.3	65.8 ± 6.8	65.8 ± 6.6	NS
Systolic BP (mmHg)	123.1 ± 15.8	122.9 ± 10.3	119.8 ± 16.8	NS
Diastolic BP (mmHg)	74.5 ± 10.5	75.2 ± 9.4	74.1 ± 12.1	NS
Adjusted calcium (mmol/L)	2.35 ± 0.07	2.36 ± 0.05	2.34 ± 0.09	NS
Phosphate (mmol/L)	1.05 ± 0.18	1.03 ± 0.18	1.06 ± 0.16	NS
Vitamin D3 (nmol/L)	48.2 ± 23.3	40.3 ± 20.6	45.6 ± 25.8	NS
PTH (pg/mL)	5.9 ± 2.1	5.8 ± 1.4	6.4 ± 2.3	NS
FGF-23 (RU/mL)	**49.7 (45.9–69.1)**	**59.1 (38.2–73.4)**	**66.6 (50.0–84.9)**	**0.028**
FGF-23% change		**−1 (−19.8–21.7)**	**19.6 (3.1–38.9)**	**0.004**
FeP (%)	**14.3 ± 3.4**	**11.4 ± 4.3**	**28.4 ± 9.2**	**<0.001**
Urinary phosphate (mmol/day)	**28.4 ± 14.7**	**23.2 ± 12.3**	**54.9 ± 19.2**	**<0.001**
Urinary cGMP (nmol/L)	472.8 (312–645.4)	530.6 (288.2–756.4	501.1 (274.9–674.0)	NS
Urinary FGF-23 (RU/mL)	46.1 (26.6–288.2)	139.5 (31.3–360.6)	227.9 (39.4–405.8)	NS
FMD post-cuff (%)	**8.4 (6.2–11.6)**	**6.6 (3.4–10.3)**	**3.4 (2.6–5.3)**	**<0.001**
FMD post-cuff % change		**−23.5 (−59 to â 0.2)**	**−58.1 (−71.9 to â 43.4)**	**<0.001**
FMD post-GTN (%)	17.7 (13.4–23.2)	17.2 (12.3–23.7)	16.3 (12.1–17.7)	NS
PWV (m/s)	7.4 ± 1.9	7.3 ± 1.7	7.1 ± 1.6	NS
AI@75	12.8 ± 12	12.5 ± 13.1	9.9 ± 12.6	NS

BMI, body mass index; BP, blood pressure; PTH, parathyroid hormone; FeP, fractional excretion of urinary phosphate; FMD, flow-mediated dilatation; PWV, pulse wave velocity; AI@75, aortic augmentation index corrected to a heart rate of 75 beats per minute; NS, not significant. P-value is in comparison with baseline values.

Statistically significant differences (P < 0.05) between baseline and subsequent measures are indicated in bold. % change refers to the % change in a particular parameter when compared with the value obtained at baseline.

**FIGURE 5:**
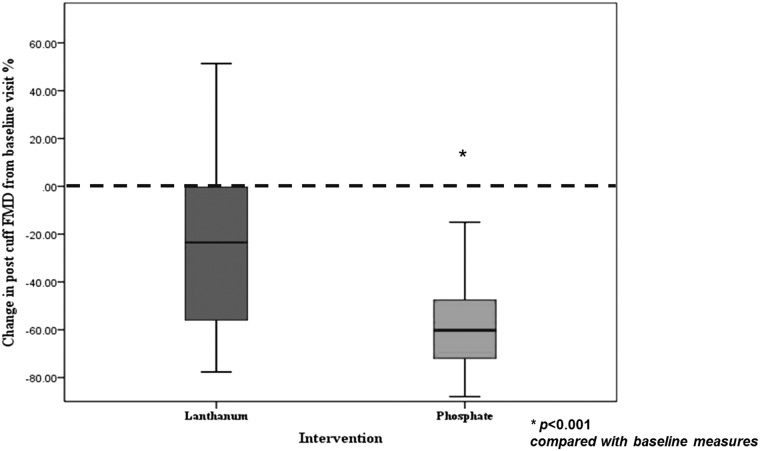
Change in FMD from baseline visit measures, following intervention with lanthanum and phosphate expressed as a % change from the baseline visit.

Table 4 shows the effect of each intervention. Phosphate supplementation was associated with a significant reduction in FMD [Figure 5; 3.4% (2.6–5.3%); P < 0.001]. With lanthanum, FMD also fell [Figure 5; 6.6% (3.4–10.3%); P = 0.033]. Post-GTN, as expected, there was significant increase in vessel diameter but there was no significant effect of phosphate supplementation or binding on GTN-mediated dilatation; randomization order was not a predictor of either FMD or GTN-mediated dilatation. In univariate analyses, the change in the serum phosphate level from baseline, change in serum FGF-23 from baseline and FeP were significant predictors of FMD. In a multiple regression model (Table 5), the inclusion of serum FGF-23 and urinary FeP was significantly associated with the change in FMD from baseline [*F*(2,54) = 10.1; P < 0.001; *r*^2^ = 0.28]. Inclusion of systolic blood pressure, serum phosphate, age, sex, urinary FGF-23, urinary calcium or urinary cGMP levels did not increase the amount of variation of change in FMD explained by the model. By recoding the three drug categories (no drug, lanthanum, phosphate), we included effects of lanthanum and oral phosphate in a multivariate model with the change in FMD as a % of the baseline visit as the dependent variable. The change in FeP and that in serum FGF-23 were included as covariates, resulting in an improved model [*F*(4,52) = 18.3; P < 0.001; *r*^2^ = 0.59].

**Table 5:** Multiple regression model with post-cuff FMD as the outcome measure

Variable	*B*	Confidence interval		P-value
		Lower bound	Upper bound	
FeP	−1.1	−1.9	−0.2	0.014
Serum FGF-23	−0.5	−0.7	−0.2	0.002

Urinary phosphate and serum FGF-23 are significant predictors of post-cuff FMD.

Urinary cGMP were measured as a marker of endothelial function, and correlated inversely with FeP, consistent with disruption of the NO pathway (*r* = −0.39; P 0.003). In univariate analysis, serum phosphate, age, male sex and FeP were associated with urinary cGMP levels. Overall, at the end of the treatment phases of phosphate supplementation and lanthanum treatment, there was no significant difference in mean serum phosphate, parathyroid hormone (PTH), vitamin D, adjusted calcium or magnesium levels compared with baseline visit values. Serum FGF-23 levels increased significantly following phosphate supplementation and fell, albeit not significantly, following lanthanum, compared with baseline.

## DISCUSSION

This paper investigates the independent, direct actions of phosphate in cells, vessels and human subjects, in order to understand the potential effects of phosphate as a CV risk factor. The prevailing view has been that elevated serum phosphate increases CV risk by promoting vascular calcification, and more recently that increased levels of FGF-23 contribute to CV risk by promoting cardiac hypertrophy. Our data provide strong support for an additional direct effect of phosphate resulting in endothelial dysfunction, mediated via disruption of the NO pathway.

### Direct actions of phosphate and the NO pathway

The *ex vivo* studies demonstrate that sustained exposure to phosphate, independent of other features of the uraemic environment, causes impaired endothelium-dependent vasodilatation in resistance vessels. This supports a direct detrimental effect of phosphate, and this appears to be mediated by NO, with evidence of reduced basal and stimulated NO production. Endothelium-independent vasodilation is also impaired in rat and human LKD vessels exposed to high-phosphate concentration, suggesting disruption of the whole NO pathway. Adding zaprinast improved both endothelium-dependent and independent vasodilatation in vessels incubated in high-phosphate conditions. Zaprinast inhibits cGMP breakdown, increasing cGMP levels, and is beneficial in pulmonary hypertension where endothelial dysfunction is present [38, 39]. This could be a potential therapeutic option.

Our cellular models support the concept of disruption to the NO pathway with reduced basal NO, reduced expression of phosphorylated and total eNOS and PKG, lower concentrations of cGMP and increased nitrotyrosine expression. Some of these effects may be reversible. The finding that CKD vessels incubated in normal-phosphate conditions relaxed as well as LKD vessels did, with carbachol is consistent with a degree of adaptability and reversibility of the effects of phosphate in CKD.

### Actions of phosphate in the uraemic environment

In CKD vessels, endothelium-dependent relaxation was impaired, but only with incubation in a high-phosphate environment. In a normal-phosphate environment, CKD vessels relax as well as LKD vessels. Unlike the LKD and rat vessels, endothelium-independent relaxation did not appear to be impaired in CKD.

These findings may reflect adaptive changes during chronic exposure to high-phosphate concentration in the uraemic environment, altering the responses to subsequent changes in phosphate. Our results conflict with a previous study from our group in human uraemic resistance vessels that showed impaired endothelium-dependent and, to a lesser extent, independent function in CKD [40]. In the present study, CKD vessels showed endothelial dysfunction, compared with normal vessels, with sustained exposure to high-phosphate concentration similar to uraemic levels. In contrast, the study of Morris *et al.* used no added phosphate [40]. It is likely that the effects of extracellular phosphate are mediated via changes in intracellular phosphate, which will equilibrate with time. Removing vessels from a CKD environment will also ‘wash out’ circulating factors, which may include asymmetric dimethylarginine and ROS [41, 42]. Phosphate itself might cause increased ROS, given that nitrotyrosine (a product of tyrosine nitration mediated by ROS) was increased in cells cultured in high-phosphate conditions. The timing of such effects and the timescale of their reversibility is unclear and requires further study.

### Physiological relevance in humans

*In vivo*, we have shown similar effects to the *in vitro* studies: sustained oral phosphate loading causes endothelial dysfunction. Serum phosphate is <1% of total body phosphate and did not change significantly during treatment. The serum FGF-23 level rose by around 20% during phosphate loading and urinary phosphate excretion by 50% compared with baseline. This suggests that compliance with study medication demonstrates phosphate homoeostasis and highlights the limitations of serum phosphate as a measure of total body phosphate. In early CKD, serum phosphate is maintained within the reference range and rises consistently only when eGFR drops below 30 mL/min/1.73 m^2^, although in population studies, serum phosphate—even within the ‘normal’ range—is linked to CV risk. It may be that serum phosphate is an insensitive, homoeostatically regulated marker of total body phosphate which, in turn, influences endothelial function. A more precise estimate of risk may be derived from serum phosphate, FGF-23 levels and urinary phosphate levels—accounting for phosphate intake and total body excess. This is supported by our findings associating elevated serum FGF-23 and urinary phosphate excretion with poorer endothelial function. FeP is likely to be a better indicator of actual phosphate leak per nephron than standard 24 h urinary phosphate, by accounting for serum phosphate levels [43]. In the clinical setting, it may be possible to use a phosphate:creatinine ratio or a spot FeP to provide additional information about phosphate intake and activation of phosphaturic mechanisms. Whether such detail has any clinical benefit would need to be investigated in a clinical trial. Several small studies have demonstrated an association between FGF-23 and dietary phosphate intake in healthy volunteers with increased phosphate intake over a few days resulting in increased levels of serum FGF-23 [44, 45]. Increased levels of FGF-23 correlate with increased serum phosphate, urinary excretion of phosphate and vitamin D levels [44]. Urinary phosphate excretion alone or in combination with measures of FGF-23 may be a better surrogate for total body phosphate than the serum phosphate level.

CKD patients may respond differently to sustained phosphate loading and thus results from our clinical study cannot be extrapolated to the CKD population. Our study was not powered to assess the direct effects of either phosphate supplementation or lanthanum and so no firm conclusions about lanthanum and endothelial function can be drawn. We chose lanthanum as a non-calcium-containing ‘single tablet’ binder, without well-described effects on confounding factors, for example the lipid-lowering properties of sevelamer [18]. Further studies on the direct effects of lanthanum on vascular function, and comparison of different classes of binder or diet, are merited.

Naturally, there are limitations of our work. We deliberately used the extreme ends of the phosphate spectrum (not least because standard concentration medium contains 0.5 mM phosphate), but dose response experiments using concentrations of phosphate in between 0.5 mM and 3 mM would be of value. We acknowledge that our findings are likely to form only part of the explanation for the increased CV risk associated with phosphate and it seems probable that vascular calcification and FGF-23 and PTH also play a role. However, to help tease out the isolated effects of phosphate, we deliberately chose to study it alone and not look at effects of its key regulators, i.e. FGF-23 and PTH *in vivo* and *in vitro.* Similarly, this is why we used healthy volunteers for our clinical study.

**FIGURE 6:**
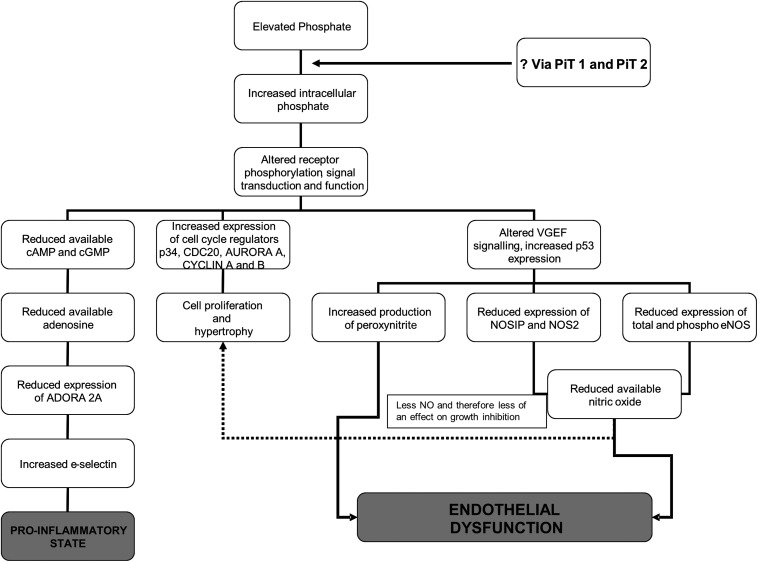
Possible mechanism of action of phosphate as a cardiovascular risk factor causing both a pro-inflammatory state and endothelial dysfunction. Using evidence presented in this manuscript and previous studies, we propose this as the possible mechanism of action of phosphate resulting in endothelial dysfunction and a pro-inflammatory state. Additionally, we have some evidence of an effect of phosphate on cell growth (data not yet published).

In conclusion, these studies show that phosphate has directly detrimental effects on the endothelium and vasculature, effects that are likely to be mediated by disruption of the whole NO pathway (Figure 6). Some of the effects may be reversible, either by manipulation of phosphate or pharmacologically, for example by inhibiting PDE5 (Figure 6). We have previously shown direct effects of phosphate on inflammation, leading to a pro-inflammatory state [46].

The clinical study presented here shows that the *in vitro* effects are of functional relevance and likely to play a role in the CV risk associated with high-phosphate concentration. It is our impression that intracellular, rather than extracellular phosphate is central to the observed effects. Drugs which inhibit cellular phosphate uptake may offer an alternative strategy to target adverse effects of hyperphosphataemia in CKD. Future work should consider the measurement of intracellular phosphate.

## SUPPLEMENTARY DATA

Supplementary data are available online at http://ndt.oxfordjournals.org.

## Supplementary Material

Supplementary Figure 1

Supplementary Table 1

Supplementary Materials and Methods
