# Supplementary figures and images for: Deleterious effects of phosphate on vascular and endothelial function via disruption to the nitric oxide pathway
Source: Nephrol Dial Transplant. 2016 Jul 22;32(10):1617–27. doi: 10.1093/ndt/gfw252 (PMC5837731; doi:10.1093/ndt/gfw252)

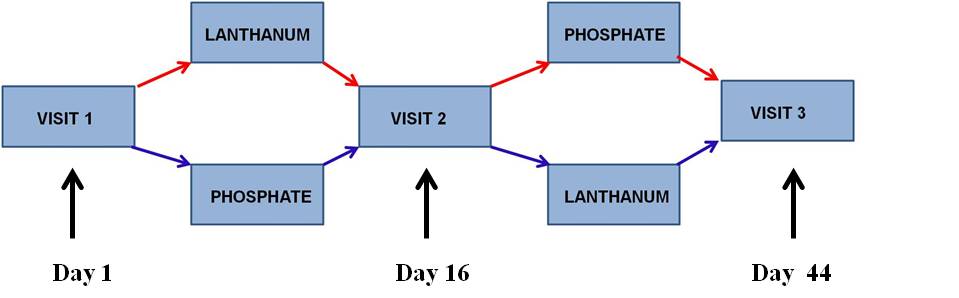

Supplement: Supplementary Figure 1 [file supplementary_figure_s1_gfw252.jpeg]
