# Supplementary material for: Deleterious effects of phosphate on vascular and endothelial function via disruption to the nitric oxide pathway
Source: Nephrol Dial Transplant. 2016 Jul 22;32(10):1617–27. doi: 10.1093/ndt/gfw252 (PMC5837731; doi:10.1093/ndt/gfw252)
Supplement: Supplementary Table 1 [file supplementary_table_1_gfw252.docx]

| **Substance** | **Normal phosphate PSS** | **High phosphate PSS** |
| --- | --- | --- |
| NaCl | 118.4 | 118.4 |
| KCl | 4.7 | 3.3 |
| MgSO_4_.H_2_O | 1.2 | 1.2 |
| NaHCO_3_ | 24.9 | 24.9 |
| KH_2_PO_4_ | 1.18 | 2.5 |
| Glucose | 11.1 | 11.1 |
| CaCl_2_ | 2.5 | 2.5 |

Abbreviations: mM, milimolar; PSS, physiological saline solution
