# Supplementary material for: Deleterious effects of phosphate on vascular and endothelial function via disruption to the nitric oxide pathway
Source: Nephrol Dial Transplant. 2016 Jul 22;32(10):1617–27. doi: 10.1093/ndt/gfw252 (PMC5837731; doi:10.1093/ndt/gfw252)
Supplement: Supplementary Materials and Methods [file materials_and_methods_supplement_ndt_gfw252.docx]

Unless otherwise stated, all chemicals and reagents were purchased from Sigma-Aldrich Ltd, Poole, UK**.**

**Vessel studies**

**Animals**

Inbred colonies of Wistar-Kyoto rats were maintained “in-house” by brother-sister mating. Microsatellite screening was used to confirm homozygosity of all loci within a random group from the strain.

12 week old male Wistar-Kyoto rats were sacrificed in accordance with the Animals Scientific Procedures Act 1986 (schedule one) and the mesentery removed and placed into a chilled normal phosphate concentration physiological saline solution (PSS). The constituents of PSS are defined in Supplementary Table 1. This was undertaken by trained staff. Prior to sacrifice, all animals were housed under controlled environmental conditions: temperature maintained at 21°C with 12 hour light/dark cycles. Rats were fed standard rat chow (rat and mouse No.1 maintenance diet, Special Diet Services) and water was provided ad libitum. The mesentery was stored in the fridge for up to one hour before it was pinned on a petri dish containing fresh physiological saline solution (PSS).

**Humans**

Patients without CKD undergoing nephrectomy for living kidney donation and patients with CKD undergoing live donor renal transplant were identified. Ethics approval was obtained prior to enrolment (West of Scotland Research Ethics Committee 4). Informed consent was obtained. Blood samples were collected the day prior to the operation and in the case of patients with CKD, prior to dialysis where possible. At the time of the operation, and prior to the use of either diathermy/harmonic scalpel, a piece of skin approximately 5mm wide and 70mm long was dissected with adherent subcutaneous abdominal fat. The sample was immediately placed into chilled PSS and transported to the laboratory

**Dissection and myography**

All vessels were dissected, on a petri dish with 5mm thick layer of sylgaard to hold fixing pins and fresh PSS, under a dissection microscope (Zeiss). Micro dissection instruments were used (World precision instruments). Dissection was performed at room temperature without oxygenating PSS. Following dissection, vessels were stored in standard PSS before being transferred to universal containers containing either normal (1.18mM) or high (2.5mM) phosphate concentration PSS. The composition of these solutions is shown in supplementary table 1. The solutions were stored at 4^o^C for 16 hours prior to use in experiments. For the human vessels, EDTA was also added to both the normal and high concentration PSS at a concentration of 0.023mM.

All myography experiments were performed on a four chamber wire myograph (multimyograph model 610, Danish Myo Technology). Experiments were conducted after storage of the vessels at 4°C in normal (1.18mM) or high phosphate (2.5mM) concentration solution for 16 hours. PSS was aerated with a 5%CO_2_/95%O_2_ mixture and had a pH of 7.4. Each chamber of the myograph was filled with PSS; two chambers contained normal phosphate and two high phosphate concentration PSS. Under a dissecting microscope, the vessels were, using micro-forceps, fed onto the wire attached to the myograph. EC50 values are expressed as the mean of the sum of the EC50s for each vessel.

**Rat vessels**

Mulvany described a process – normalisation - by which to standardise third order rat resistance vessels on a myography. ^1^ Normalisation establishes the internal circumference that a vessel would have when relaxed under a trans-mural pressure of 100mmHg (L_100_). This is undertaken by gradually distending the vessel using the micrometer and recording the micrometer and force readings. The Laplace equation is then used to calculate the L_100_. The internal circumference is then set to 90% of the L_100_ .^2^

Following normalisation, the vessels were subjected to a ‘waking up’ protocol where KPSS (PSS with potassium chloride substituted for sodium chloride on an equimolar basis) was added to the bath and the vessels contracted. The vessels were then contracted with PEP and relaxed with carbachol, in a stepwise fashion. Vessels which failed to contract to KPSS or PEP or failed to relax >50% to carbachol were discarded. The vessels were then left for 30 minutes prior to concentration response curves being calculated. Vessels were contracted with PEP in the presence and absence of L-NAME (1x10^-5^M for one hour). Following contraction with PEP, they were relaxed with carbachol or with SNP. Vessels were also contracted and relaxed in the presence of zaprinast, a phosphodiesterase-5-inhibitor (PDE5I) (Tocris bioscience, Bristol, UK). Incubation with zaprinast was for one hour.

**Human vessels**

Mulvany’s process of normalisation refers specifically to third order rat resistance vessels and there is debate as to the applicability to other types of vessel. ^3^ Therefore, for the human resistance vessels, active and passive tension curves were constructed as described by Van den Akker ^3^. The force on the vessels was standardised at the point where the constructed active and passive tension curves crossed: 0.8g of tension. Approximately 0.2g of tension was added to the vessels every 15 minutes to reach 0.8g. The vessel diameter was measured manually by taking the micrometer reading when the wires were just touching and subtracting this from the reading when the wires were touching the vessel wall and adding 80 microns (wires 40microns). The vessel length was measured. Vessels were ‘woken’ with KPSS. They were contracted with PEP to achieve a maximal response. After wash out the vessels were contracted with PEP to 60% of the maximum response and relaxed with carbachol. Following a wash out period, the vessels were contracted again with PEP and relaxed with SNP. Only vessels which did not contract to KPSS were discarded.

**Statistical analysis**

All responses are expressed as mean ± SEM and comparison made between AUC of groups with the Student’s T test, unless otherwise stated. For comparisons between maximal vasodilation or contractile responses, an unpaired Student’s T test (rat mesenteric vessels) or an ANOVA with Tukey’s post hoc analysis (human resistance vessels) was used. The median L100 (rat vessels only) and the median vessel lengths were compared with a Mann Whitney U test. Statistical analysis was performed in SPSS v 19 (IBM, Armonk, New York) and results are expressed as mean ± SEM.

**Cell culture**

HUVECs (Promocell, Heidelberg, Germany, catalogue number: C-12203) and rat myoblasts (ATCC, Rockville, ND, catalogue number: CRL-1476) were used. We exposed cells and vessels to two phosphate concentrations, normal and high (0.5mM and 3mM in cell lines and 1.18mM and 2.5mM in vessels). For context, 0.5mM is the standard concentration of phosphate in the conventional cell culture medium suited to the cells utilised, 0.8-1.4 mM is the normal plasma range in humans whilst 2.5-3mM is at the extreme of the CKD spectrum.

All cultures were incubated at 37°C in 5% CO2. Appropriate cell media was either standard phosphate concentration media (0.5mM) or custom formulated with a phosphate concentration of 3mM from PromoCell (catalogue numbers: C-22062, C-97109, C-39267, C-22215, C-97084, C-39215). On arrival, cells were grown in standard phosphate concentration medium until they reached 90% confluence. At the first passage in our laboratory, they were divided into normal phosphate (0.5mM) and high phosphate (3mM) concentration cells and grown in the appropriate phosphate concentration media from that point until they were discarded. HUVECs were used between the second and fifth passages and for each different experiment, the cells were used at the same passage. Every experiment was repeated a minimum of 4 times.

**Clinical Study**

The West of Scotland Research Ethics Committee approved this study prior to enrolment and patients provided informed, written consent.

This was a single blind, cross-over study. Each participant attended (fasted) for three visits. At visit one, patients were randomised to receive either one 500mg phosphate tablet or 1000mg lanthanum carbonate tablet three times daily for two weeks. The participant knew which tablet they were taking but the investigator did not. After two weeks, patients attended for visit two. After a further two week wash out period, patients received the other drug for two weeks, before attending for the final visit. Supplementary Figure S1 online illustrates the study visit protocol. At each visit, the same measures were taken and pill counts were performed to assess compliance at visits 2 and 3

Serum and plasma samples were sent to the laboratory and additional blood was stored at -80°C for FGF-23, cGMP and vitamin D analysis. Prior to each visit, a 24 hour urine collection was obtained. Urine was analysed for electrolytes using flame photometry. Aliquots of urine were stored at -80°C for FGF-23 and cGMP analysis.

Endothelial function was measured using FMD and measurements standardized. (44) All recordings and analyses were performed by one investigator (8.0MHz linear array transducer (Acuson 8L5 system, Sequoia 512, Siemens). Vascular stiffness was measured using SphygmoCor® Vx system (Atcor Medical, Sydney, Australia) and standardised. (45)

**Statistical analysis**

Data were assessed for normality and log transformed where appropriate. Uni and multivariate linear regression models were constructed with outcome (change in FMD from baseline) fitted as a function of treatment, patient and period but in such a way that the relevant contrasts could be specified. One third of the images were randomly selected and re-analysed to test reproducibility and intra-observer variability.

**cGMP measurement**

This was measured according to the manufacturer’s instructions using an ELISA kit (Cell Signaling technology, distributed by New England Biolabs Ltd, UK). Analysis was by Mann Whitney U test in GraphPad Prism, CA, USA.

**Cell culture**

### Griess reaction

Cells were plated onto a 96 well plate. After 24 hours fresh media was added to each well. Simultaneously cells were treated with L-NAME (1x10^-4^M) or L-Arginine (1x10^-5^M); controls were left untreated. L-NAME was used as a NOS inhibitor and thus a reduction in NO would be expected. L-arginine was added to increase the available substrate for eNOS with an anticipated subsequent increase in NO production. The media was replaced daily with fresh L-NAME or L-arginine as appropriate until day four when a Griess reaction was performed. Sodium nitrite was used to generate a standard curve in each plate at a concentration of 0-100µM. 50µl of Griess was added to each well, the plate was then wrapped in tinfoil and replaced in the incubator for 30 minutes before being read at an absorbance of 540nm on a microplate reader. This was performed in duplicate and a minimum of six replicates were used for each condition.

### Preparation of cell lysates

Supernatant was removed from cells and either discarded or stored in Eppendorf tubes at

-80°C. On ice, cells were washed three times in ice cold PBS, before 70µL of radio-immunoprecipitation assay buffer (RIPA) was added to individual wells. RIPA was made in house (150mM NaCl, 1mM EDTA, 1% (v/v) TX100, 0.5% (w/v) deoxycholic acid and 50mM Tris pH7.4; concentrations are final concentrations)and stored at 4°C. Prior to use, RIPA was supplemented with the protease inhibitors benzamidine and soya bean trypsin inhibitor, sodium orthovanadate and PMSF (phenylmethanesulphonyl fluoride). A pipette tip was used to scrape the adherent cells and the plate were placed in the freezer (-20°C) for 20 minutes. Lysates were collected after thawing and stored at -80°C until use. Repeated freeze-thaw was avoided.

### Quantification of protein concentration

The Bradford assay was used to quantify protein concentration. BSA standards were used to construct a standard curve at a concentration of 0-0.225µM. 0.5ml of Bradford reagent (BioRad Laboratories, Germany) was added to a cuvette containing 5µL of sample. The samples were read using a spectrophotometer (Beckman DU 640D) at an absorbance of 595nm. The standards and samples were measured in duplicate and an average taken.

### Polyacrylamide gel electrophoresis and immunoblotting

Proteins were resolved using the NuPAGE 4-12% Bis-Tris pre-cast gel system (Life technologies, Paisley, UK formerly Invitrogen). Minimum sample protein content was 10ug per well. Protein samples were denatured in NuPAGE LDS (lithium dodecyl sulphate at a pH of 8.4) sample buffer (Life technologies, Paisley, UK) by heating to 70°C for 10 minutes. In order to reduce proteins, 50mM DTT was added immediately prior to sample denaturing. Proteins resolved by polyacrylamide gel electrophoresis were transferred to Protran nitrocellulose membranes using the NuPAGE XCell II blotting apparatus and the manufacturer’s transfer buffer which was supplemented with 20% (v/v) methanol. Transfer was performed at 40V for 60mins. Transfer efficiency was assessed by staining membranes with Ponceau S solution followed by washing with TRIS buffered saline (TBS) (20mM Tris-HCl pH 7.4, 135mM sodium chloride sterilized by autoclave) containing 0.1% (v/v) Tween 20 (TBST). The nitrocellulose membranes were then blocked in either 5% (w/v) non-fat powdered milk (Marvel) or 5% (w/v) BSA for 1 hour at room temperature. Blots were incubated with primary antibodies at the optimal concentration in either milk or BSA overnight at 4°C. Five washes of five minutes with TBST were then carried out prior to incubation with the appropriate horseradish peroxidise conjugated secondary antibody for two hours in milk or 5% (w/v) BSA at room temperature. Blots were then washed with TBST for five minutes five times. Proteins were visualised by enhanced chemiluminescence (Amersham, GE Healthcare, UK) and autoradiography. Phospho and total eNOS antibodies were purchased from Cell Signaling Technology (catalogue numbers: #9571 and #9586), nitrotyrosine from R&D systems (catalogue number: MAB3248), PKG from Enzo life sciences (catalogue number: ADI-KAP-PK002), GAPDH from Abcam (catalogue number: 9484) and the secondary antibodies from Amersham (catalogue numbers: NA9310 and NA9340).

### Epi-fluorescence and FURA -2-AM

Intracellular calcium concentration was measured in HUVECs and rat VSMCs with epifluorescence using a dual-wavelength spectrophotometric method and the fluorescent dye FURA-2-AM (FURA-2-acetoxymethyl ester).

FURA-2-AM is a membrane permeable derivative of the ratiometric calcium indicator, FURA-2. FURA-2-AM crosses into a cell through the membrane and the acetoxymethyl groups are cleaved by cellular esterases; this process regenerates FURA-2, the pentacarboxylate calcium indicator. Cells imaged under fluorescence can be measured at two wavelengths, 340nm and 380nm. Intracellular calcium concentration can then be calculated based on 340/380 ratios. Using the ratio automatically cancels out variables which may lead to artefact when imaging intracellular calcium concentration; including local differences in FURA-2 concentration and variations in cell thickness. ^4^ The intracellular calcium value (R) which corresponds to a particular 340/380 ratio is calculated with this formula: ((R-R_min_)/(R_max_-R))*1.2x10^-6^M 1.2x10^-6^M is the dissociation constant of FURA-2-AM. The minimum ratio (R_min_) is the fluorescence obtained using a zero calcium solution (10mM EGTA). The maximum calcium ratio (R_max_) is the fluorescence ratio obtained when the cell is lysed, thus releasing all of the intracellular FURA-2.

Cells were plated in glass bottomed plates and allowed to adhere overnight. The following day, cytosolic loading of FURA-2-AM (Life technologies, Paisley UK, formerly Invitrogen) was achieved by incubating the cells with FURA-2-AM (10µM endothelial cells and 5µM SMCs) at 37^o^C for 40 minutes (ECs) or 15 minutes (VSMCs). The cells were then washed with 1ml of media and incubated at 37^o^C for a further 15 minutes. Plates were mounted on an inverted microscope and isolated cells were imaged. Using an oscilloscope and the Clampex data acquisition software (pCLAMP, Molecular Devices LLC, USA), baseline fluorescence was recorded. 2µM calcium ionophore (non-fluorescing form, A23187) was added to prove that a calcium sensitive signal could be generated. On addition of calcium ionophore, the 340/380 fluorescence ratio rises and plateaus. Following this the cell lyser, saponin was added (10µM) to trigger cell lysis. A recording was made until the 380 nm wavelength began to fall. The Clampfit programme (Molecular Devices LLC, USA) was used to analyse the data and to determine the baseline fluorescence and the R_max_ values.

To calculate the R_min_ of the cells and the dissociation constant (Kd) of FURA-2-AM, a three point calibration curve was constructed using HUVECs. Cells were plated in three dishes as described above and then incubated with 10µM FURA-2-AM for 40 minutes at 37^o^C. The cells were washed and incubated for a further 5 minutes. 2µM calcium ionophore was added and the plates were left at 37^o^C for a further 2 minutes. A zero calcium HEPES (100mM KCl, 1mM MgCl_2_, 25mM HEPES, 10mM NaCl) solution was used to wash the cells and then a 10mM EGTA HEPES solution was added to one plate, a 5mM/5mM calcium EGTA/HEPES solution was added to the 2^nd^ and to the 3^rd^ a 10mM calcium EGTA solution was added.

The plates were incubated for 10 minutes at 37^o^C. The fluorescence ratio was measured on the oscilloscope. The free calcium concentration of each of the three added solutions was calculated using REACT software (10nM, 37.5µM and 61.7µM respectively). The free calcium concentration was then plotted on a log scale on the X axis and the fluorescence ratio (R_min_) recorded for each solution was plotted on the Y axis. A sigmoidal curve was drawn. The 50% value is equivalent to the Kd of FURA-2-AM and the R_min_ value for the cell line is equivalent to the fluorescence ratio seen with the 10mM EGTA solution.

**Clinical Study**

**Measurement of stored samples**

All stored samples were measured after a single thaw. 25-hydroxyvitamin D2 and D3 concentrations were measured by liquid chromatography-tandem mass spectrometry (LC-MS/MS) using a Waters UPLC with the ACQUITY TQD. The current assay Coefficient of variation is D3 mean 14nM CV 8.9%, mean 52.7nM CV 7.4%; D2 mean 14nM CV 9.8%, mean 47.7nM CV 8.8%.

FGF-23 was measured on EDTA plasma samples by ELISA (Immunotopics, Inc. San Clemente, CA). The ELISA was performed in accordance with the manufacturer’s instructions. Urinary FGF-23 was measured with the same ELISA kit.

Urinary cGMP was measured by ELISA (R&D systems Europe Ltd. Abingdon, UK) on samples from a 24 hour urine collections. Samples were diluted 10 fold using the calibrator dilutant (RD5-5) supplied with the kit.

**FMD**

The brachial artery was identified and scanned longitudinally approximately 10 cm proximal to the antecubital fossa. Within the brachial artery, clear vascular boundaries were identified to allow imaging of the double lines of Pignoli ^5^ and this in turn allows a more precise diameter measurement (≤0.05 mm) to be calculated by automated edge-detection software (Vascular Research Tools package version 5, Medical Imaging Applications LLC, USA). ^6^ Each study was recorded and analysed with automated edge detection software from Medical Imaging Applications LLC. This type of software is known to reduce intra-observer variability, improve reproducibility and the validity of FMD measures. ^6^ FMD was expressed as the percentage change in the arterial diameter pre and post cuff occlusion, relative to the baseline diameter. For endothelium independent measures following GTN, the change in arterial diameter was expressed as the percentage change in the arterial diameter pre and post GTN spray, relative to the 2^nd^ recorded baseline diameter.

#### Randomisation, power calculation and statistical analysis

Dr Patrick Mark (Senior Clinical Lecturer, University of Glasgow) generated a randomisation list in Microsoft Excel. This was given to the Pharmacy Preparation Unit at the Western Infirmary who put the drugs into an inner box and an outer bag. The inner box was labelled with details of the drug and the outer bag with participant identifiers but no drug identifiers. The Western Infirmary pharmacy dispensed the drugs.

Statistical advice with regards to sample size and power calculations was taken from an independent statistician, Professor Stephen Senn (formerly of The School of Mathematics & Statistics, University of Glasgow). We anticipated that FMD measures would differ between the highest and lowest phosphate measures. For a clinically relevant difference of 3% in FMD, and a within-patient standard deviation of three (which corresponds to a standard deviation for the difference between repeated measures of about 4.25) approximately 18 patients would be needed to have 80% power for a 5% significance level (two sided). The aim was to recruit between 18 and 24 patients, to be randomised in equal numbers, per sequence. This would provide 90% power should all patients complete but allowed some margin in the event of drop-out. In fact 20 patients were recruited and randomised in equal numbers, 10 per sequence.

Results for urinary phosphate are presented as the fractional excretion of phosphate calculated as follows:

(Urinary phosphate * Serum creatinine) / (Urinary creatinine * Serum phosphate) %

By taking into account urinary creatinine, this is more likely to be an accurate reflection of true urinary phosphate excretion.

Reference List

(1) Mulvany MJ, Aalkjaer C. Structure and function of small arteries. *Physiol Rev* 1990;70:921-961.

(2) Slezak P, Waczulikova I, Balis P, Puzserova A. Accurate normalization factor for wire myography of rat femoral artery. *Physiol Res* 2010;59:1033-1036.

(3) van den Akker J, Schoorl MJ, Bakker EN, Vanbavel E. Small artery remodeling: current concepts and questions. *J Vasc Res* 2010;47:183-202.

(4) Eisner DA, Nichols CG, O'Neill SC, Smith GL, Valdeolmillos M. The effects of metabolic inhibition on intracellular calcium and pH in isolated rat ventricular cells. *J Physiol* 1989;411:393-418.

(5) Pignoli P, Tremoli E, Poli A, Oreste P, Paoletti R. Intimal plus medial thickness of the arterial wall: a direct measurement with ultrasound imaging. *Circulation* 1986;74:1399-1406.

(6) Woodman RJ, Playford DA, Watts GF et al. Improved analysis of brachial artery ultrasound using a novel edge-detection software system. *J Appl Physiol* 2001;91:929-937.

| **Substance** | **Normal phosphate PSS** | **High phosphate PSS** |
| --- | --- | --- |
| NaCl | 118.4 | 118.4 |
| KCl | 4.7 | 3.3 |
| MgSO_4_.H_2_O | 1.2 | 1.2 |
| NaHCO_3_ | 24.9 | 24.9 |
| KH_2_PO_4_ | 1.18 | 2.5 |
| Glucose | 11.1 | 11.1 |
| CaCl_2_ | 2.5 | 2.5 |

Abbreviations: mM, milimolar; PSS, physiological saline solution

**Table S1: The concentration (mM) of the constituents of normal and high phosphate concentration solutions used in the myography experiments.**

*For the human vessels, EDTA was also added to both solutions, at a concentration of 0.023mM*


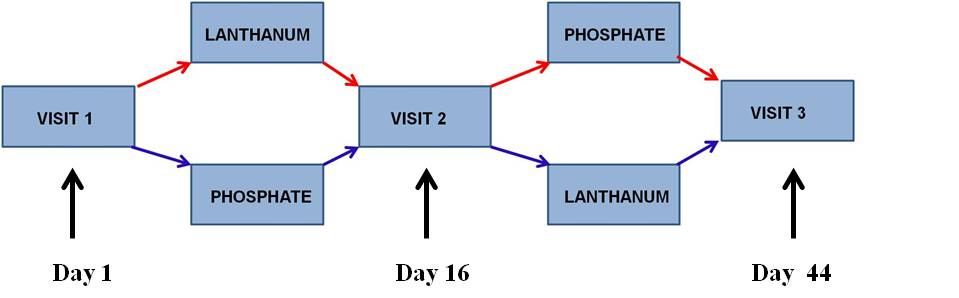


Figure S1: Study Protocol.

*Volunteers attended for a baseline visit followed by two further visits. Prior to visit two, volunteers took either lanthanum carbonate or phosphate supplements and after a wash out period took the other drug before attending for the final visit. Patients were randomised at visit one to follow arm one (red arrows) or arm two (blue arrows)*.
